# Facile assembly of flexible quaternary SnO_2_/SrSnO_3_/Fe_3_O_4_/PVDF nanocomposites with tunable optical, electrical, and magnetic properties for promising magneto-optoelectronic applications

**DOI:** 10.1038/s41598-023-32090-w

**Published:** 2023-03-27

**Authors:** A. M. Ismail, Fawzy G. El Desouky

**Affiliations:** 1 Spectroscopy Department, Physics Research Institute, National Research Centre, Cairo, 12622 Egypt; 2 Solid State Physics Department, Physics Research Institute, National Research Centre, Cairo, 12622 Egypt

**Keywords:** Physics, Applied physics, Condensed-matter physics, Electronics, photonics and device physics, Optical physics

## Abstract

Facile assembly, co-precipitation, and drop casting procedures have been used to construct SnO_2_/SrSnO_3_/Fe_3_O_4_/PVDF flexible nanocomposites. SnO_2_/SrSnO_3_/Fe_3_O_4_ nanocomposites (TSF NCs') have been successfully incorporated into polyvinylidene fluoride polymers (PF), according to the microstructural exploration of the systems, which was revealed by XRD, EDX, and ATR-FTIR analysis. The FESEM and cross-section areas demonstrated that the addition of TSF NCs' to PF porous material enhanced its surface characteristics and decreased its surface roughness. The optical gap was lowered from 3.90 to 3.07 eV, and it was discovered that both the refractive index and optical conductivity had improved when TSF NCs' were incorporated into PF. According to the observations, the supplement ratios have a profound influence on the dielectric properties of the nanocomposites. Moreover, the electrical parameters of TSF/PF nanocomposite are significantly modified. The TSF/PF magnetic nanocomposite has good magnetic reactivity and can be easily extracted from the aqueous solution using an external magnetic field, as demonstrated by VSM. This research has been conducted to obtain TSF/PF nanocomposites to be used in promising magno-optoelectronic applications.

## Introduction

Polymeric materials are used instead of traditional materials because they are cheap, light, and have the proper physical and chemical properties^1,2^. The performance properties of polymer composites are superior to those of individual polymers. It is simple to modify the microstructural, electrical, mechanical, and other properties of polymers by adding nanofillers into the polymer matrices in varying amounts^3,4^. Modified polymer composites are significantly affected by the size, shape, concentration, and interfacial contact with nanoparticles. The strength of the bonds between polymer and nanoparticles is an important consideration for composite material performance^5,6^. Ferroelectric polymers based on poly(vinylidene fluoride) (PF) typically have a high ε_r_ value due to the existence of strongly electronegative fluorine as well as the momentary orientation of CF dipoles in crystallized processes. Owing to their analogous electrical properties with the matrix, fluoro-polymer shells have been proven to be efficient in lowering dielectric loss and improving filler dispersion^7^.

Understanding the ferroelectric polymer shell layer for composite characteristics and demonstrating the beneficial impact on enhancing filler dispersion are both important outcomes of the alteration of inorganic fillers by the ferroelectric polymer PF film^8^. Numerous future technological applications will require novel physical and chemical features, which nanocomposites with two or more separate functions can demonstrate. Improved energy conversion and storage technologies are just one way in which nanomaterials play a crucial role in the age of the smart world. Nanotechnology's ability to shrink the size of materials while maintaining or even improving their properties has had a profound impact on people's daily lives, particularly through its ability to improve the efficiency of devices such as lithium-ion batteries, electrochemical supercapacitors, solar cells, water purifiers, and electrochemical sensors^9^. The size dependence of the electrical structure of semiconducting metal oxide has attracted a lot of research. By adjusting the size and complexity of individual components, it provides a versatile design for a wide range of devices and systems with specialized electrical properties^10^. In addition, numerous methods, such as changing the lattice planes, adding surface defects, expanding the active site, and creating the lattice mismatch through doping, can boost changes in the characteristics of metal oxide^11^. Tin oxide (SnO_2_) is an n-type semiconductor that possesses a high optical gap (3.6–4.0 eV) and has a variety of applications. Some of these applications include secondary lithium batteries, solar cells, gas sensors, and glass electrodes. It has a structure known as tetragonal rutile, and the inherent oxygen vacancies in this structure operate as an n-type dopant^12–14^. Strontium stannate (SrSnO_3_) has found use in a variety of industries, including high-temperature nitrogen oxide sensors, lithium-ion batteries, and high-temperature humidity sensors, amongst other applications. This perovskite semiconducting material has a relatively large band gap (4.1 eV), which implies that it can only absorb light in the UV area^15^. This is the primary disadvantage of this semiconducting material. It has been feasible to produce materials with intriguing structural, magnetic, and semiconducting properties by loading SrSnO_3_ with transition metals and rare earth elements^16,17^. In-depth research and development have recently been concentrated on nanosized magnetic composite materials with controllable compositional, optical, electric, magnetic, and surface properties. Dimensionality and the proximity effect with nearby materials are two primary principles that can be used to engineer the qualities of a composite material beyond its chemical composition and structure^18^. Magnetite (Fe_3_O_4_), alternatively, is a metal oxide magnet that occurs naturally and is the magnetic mineral that occurs in the greatest abundance on earth. It is considered that magnetite would be ideal filler because it is readily available, inexpensive, and generates a significant amount of free energy upon reaction^19^. Recently, Prathiba et al.^20^ studied the effect of Fe-doped SrSnO_3_ on the structural, magnetic and semiconducting properties of SrSnO_3_. Paranjape et al.^21^ SnO_2_/PF composite materials with dopamine. Huang et al.^22^ magnetite/GO composite nanoparticles are assembled in an organized sequence by a magnetic field to create magnetite/GO/PF. Karim et al.^23^ magnetite/SnO_2_/RGO nanocomposites are created for supercapacitors and photocatalysts that respond to visible light.

In this study, we created an integrated and simple synthesis assembly method. This approach is the first to prepare a nanocomposite with SnO_2_/SrSnO_3_/Fe_3_O_4_ (TSF NCs) as filler in a PF matrix. Structural, optical, surface morphology, dielectric, and magnetic properties of prepared polymer nanocomposites are discussed in order to better understand structure-performance relationships in optical, dielectric, and magnetic properties, which could be extended to magneto-optoelectronic devices in the meantime.

## Materials and methods

### Materials

Sodium tin(IV) oxide trihydrate(Na_2_SnO_3_·3H_2_O), (Alfa Aesar 98%), Strontium acetate ((CH_3_CO_2_)_2_Sr (Sigma Aldrich98%), ferric chloride (FeCl_3_·6H_2_O), ferrous sulphate (FeSO_4_·7H_2_O), sodium hydroxide (NaOH), Sigma Aldrich 99%)**,** polyvinylidene fluoride (PF) with M.W = 572,000(Alfa Aesar). Dimethyl Formamide (DMF) was supplied from SD fine chemicals. All of the precursors being used are AR grade and were provided without any further purification.

### Preparation of SnO_2_/SrSnO_3_/Fe_3_O_4_ nanocomposites (TSF NCs')

#### Synthesize of SnO_2_/SrSnO_3_

In the first procedure, 30 ml of deionized water was utilized to solubilize 0.03 mol of sodium stannatetrihydrate salt as solution A, and 30 ml of deionized water was used to disperse 0.01 mol of strontium acetate bulk as solution B. A magnetic stirrer was then employed to stir the mixture continuously for 6 h. The clear NaOH solution was then added gradually to the translucent solutions of A and B. The product was separated and allowed to air dry for 24 h at 80 degrees Celsius. The finished product was annealed at 650 °C for 4 h in the air.

#### Synthesize of SnO_2_/SrSnO_3_/Fe_3_O_4_ nanocomposites (TSF NCs')

The first process involves dissolving bulk ferrous sulphate (FeSO_4_·7H_2_O) and ferrous chloride (FeCl_3_·9H_2_O) in 30 ml of deionized water each; stirring constantly with a magnetic stirrer for four hours to produce the translucent solutions. The second stage is to raise the pH to 12 by adding a clear NaOH solution. The combination was at that time continuously stirred for five hours by a stirrer.After collecting the precipitate and centrifuging it numerous intervals in ethanol and deionized water to obtain the simple solution, it was vacuum dried for 12 h at 368 K. SnO_2_/SrSnO_3_/Fe_3_O_4_ nanocomposites with a ratio of (1:0.4) of SnO_2_/SrSnO_3_ and Fe_3_O_4_ have been produced using the wet chemical co-precipitation process and labeled as TSF. The first and second phases are the prepare of annealed SrSnO_3_ and Fe_3_O_4_ phases, as indicated in sectors 2.1.1. The third stage is to make nanocomposites of SnO_2_/SrSnO_3_ and Fe_3_O_4_. A conventional process includes dispersing weight proportions of produced STO and FO products in 50 ml of deionized water and stirring constantly for two hours using a magnetic stirrer. The precipitate was vacuum dried for 12 h at 368 K after being collected and centrifuged more than once in deionized water and ethanol to achieve the basic solution.

### Preparation of TSF NCs'/PF nanocomposites

**Figure 1:**
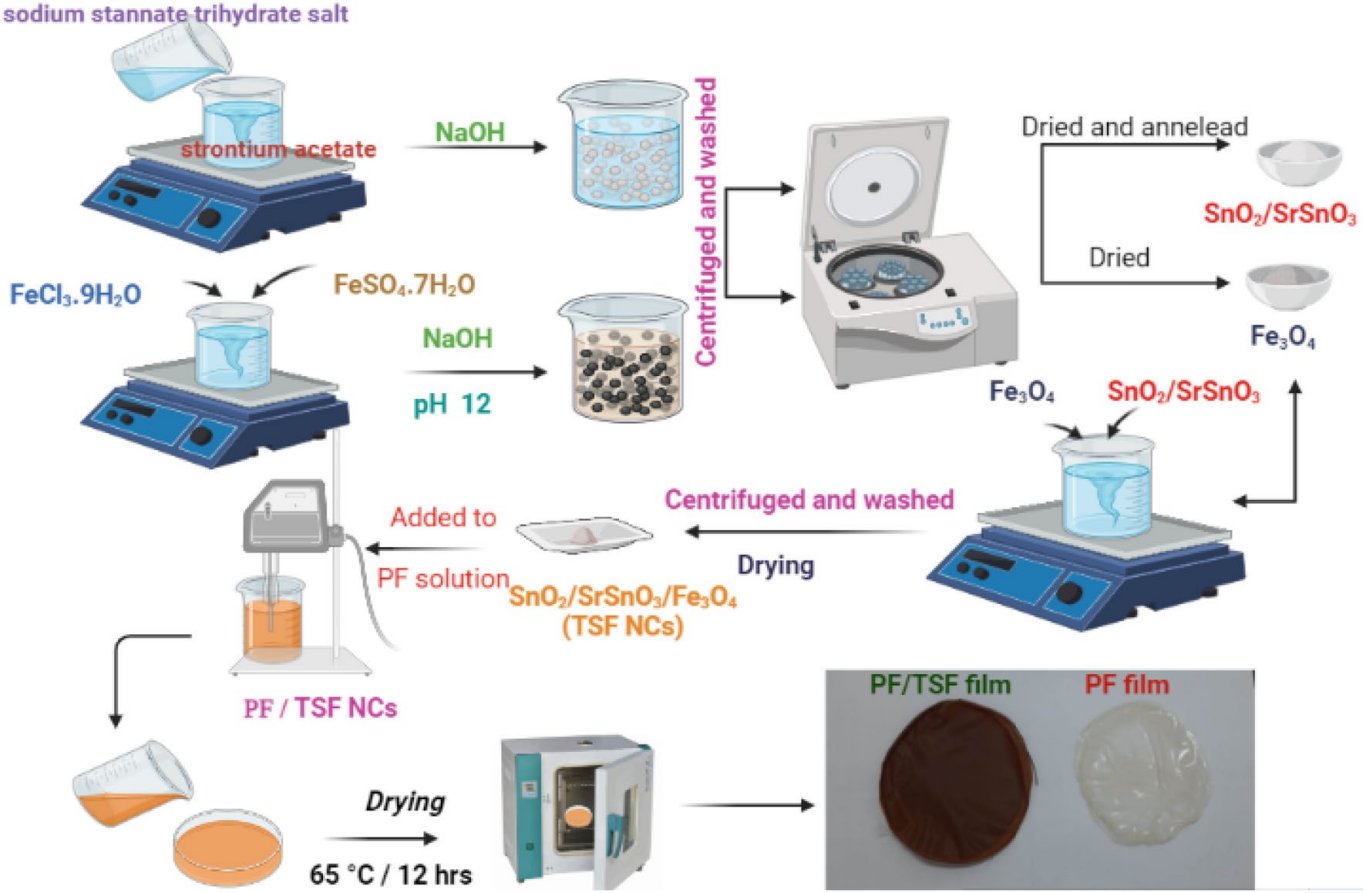
Schematic illustration of experimental work.

To begin, an optimum amount of PF powder was suspended in DMF at 65 °C till the comprehensive solubilization, and afterwards TSF NCs with various weight percentages (10, 20, and 30 wt%) were introduced to the PF solution. Then a nocomposite solution was then sonicated with a dip sonicator to prevent nanoparticle agglomeration. Finally, the solution was put into a Petri dish and left in the dryer at 65 °C for approximately 12 h. Experimental steps are represented in Fig. 1.

### Measurement techniques

PANalyticalX'Pert Pro target Cu-K with secondary monochromator Holland radiation was used to measure X-ray diffraction (XRD) data in the range of 2 from 5° to 80° utilizing tube running at 25 kV and wavelength = 0.1540 nm wavelength. Attenuated total reflectance Fourier transform infrared (ATR-FTIR) was investigated using Bruker Vertex 80. Field emission scanning electron microscopy (FESEM, Quanta 250 FEG FEI) with EDX detector was used to study the surface morphology of PF/TSF samples. High-resolution transmission electron microscope (HRTEM) was performed by JEM-2100F electron microscope with accelerating voltage of 200 kV. The UV–Vis spectrophotometer measured the UV–Vis absorption spectra from 190 to 1000 nm (Jasco V-630, Japan). Broad band dielectric spectroscopy (type concept 40) novocontrol high-resolution alpha analyzer with Quatro temperature controllers was used to get the dielectric data and the AC conductivity. Measurements of magnetic properties were taken with a vibrating-sample magntometer (VSM) series instrument (Lake shore VSM 7410).

## Results and discussions

### Structural and morphology characterizations

**Figure 2:**
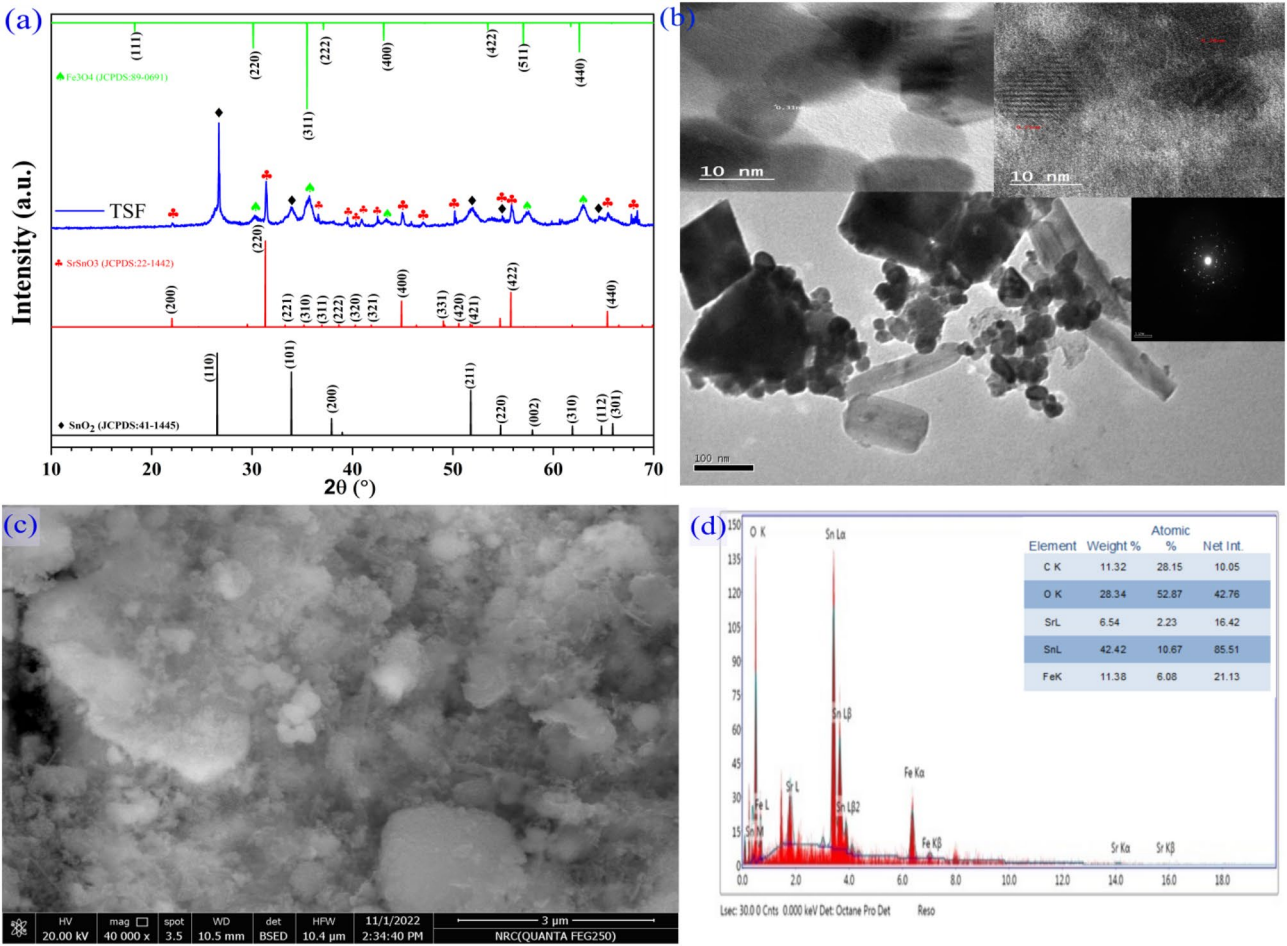
(**a**) XRD pattern, (**b**) HRTEM images, (**c**, **d**) FESEM micrograph, and (**d**) EDX of TSF NCs'.

As illustrated by Fig. 2, the prepared TSF NCs' structural, morphology, and composition were analyzed using the XRD spectrum, TEM, FESEM, and EDX graphs. Figure 2a illustrates the XRD pattern of TSF NCs, which all had prominent lines at two theta of 26.68°, 33.94°, 51.91°, and 62.94°, which match up to the standard card (JCPDS: 41–1445) of tetragonal nanostructured tin oxide^24^,and a spectra lines at two theta 22.04°, 31.34°, 39.23°, 40.89°, 42.5°, 44.96°, 47.0°, 50.45°, 51.87°, 55.84°, 57.31°,and 65.43° concerning the cubic phase of strontium stannate that matches up to JCPDS (22-1442), which typically refers to the results of recently reported works^25^,Also a spectra lines at two theta 30.28°, 35.68°, 43.32, 47.16°, 57.31°, and 62.94° could be understood in the context of Fe_3_O_4_, cubic configuration (JCPDS No. 89-0691)^26^ providing a combination of these three phases. There are no diffraction lines from other substances, indicating the high purity of the constructed sample.As mentioned in formula (1), the Scherrer model was applied to establish crystallite size (R) based on the most characteristic peaks along (110), (220), and (311) of tin oxide, strontium tin oxide, and magnetite to be 75, 47.68, and 11.3 nm, respectively^27^.

1 $$R = {\raise0.7ex\hbox{${0.94}$} \!\mathord{\left/ {\vphantom {{0.94} {\beta Cos\left( {\theta } \right)}}}\right.\kern-0pt} \!\lower0.7ex\hbox{${\beta Cos\left( {\theta } \right)}$}}$$

where λ is defined as the X-ray wavelength. β is defined as the full width at half maximum and θ is defined as the Bragg angle. Moreover, the HRTEM images in Fig. 2b clearly demonstrate elongated nanorods, nanocubes, and nanoparticle-like morphological shapes, and even the selected area of the diffraction pattern has shown a large number of bright spots that suggest the polycrystalline existence of nanocomposites, as illustrated by an inset in Fig. 2b. Additionally, TSF NCs' depict well-known lattice fringes; the gap among nearby lattice planes has a width of 0.31 nm, corresponding to the (110) plane of the rutile tetragonal structure of tin oxide, 0.28 nm related to the (220) plane of the cubic structure of SrSnO_3_, and 0.25 nm associated with the (311) plane of the spinel cubic structure of Fe_3_O_4_, as shown as an inset in Fig. 2b, that agree with the XRD data. As shown in Fig. 2c, the FESEM micrograph characterizes the surface morphology of nanocomposites, which exhibits the propagation of nanorods and nanoparticles on the surface. The elemental composition in Fig. 2 provided as an inset from the EDX spectrum. The examined mass spectra of Sn, Sr, Fe, and oxygen were attributed to the formation of TSF NCs, as well as the appearance of minor mass ratios from carbon due to chemical preparation processes and the sample holder.

**Figure 3:**
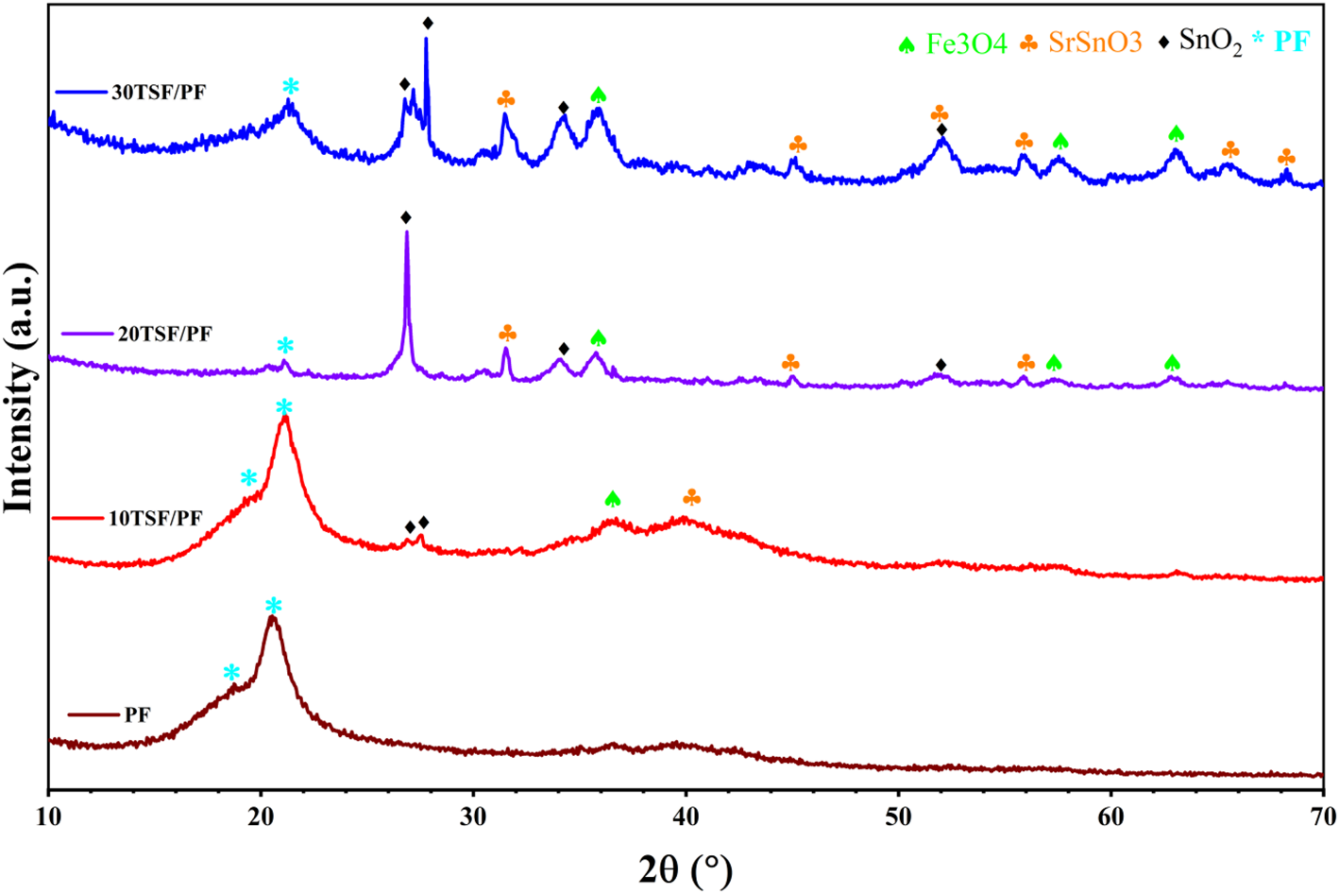
XRD spectra of PF, 10TSF/PF, 20TSF/PF, and 30TSF/PF nanocomposites.

The amalgamation of ternary SnO_2_/SrSnO_3_/Fe_3_O_4_ nanocomposites in the PF matrix template with various mass ratios has been evidenced by XRD exploration, as revealed in Fig. 3. Two observable diffraction characteristic peaks for neat PF are at 18.20° and 20.44° in relation to diffractions in planes (0 2 0) and (1 1 0) of PF, which would have been a phase peculiarity^28,29^.

The addition of TSF NCs' had no obvious effect on the phase of PF, as shown in Fig. 3 at the ratio of 10 TSF/PF. The diffraction peaks of TSF NCs' were clearly visible as the proportions were increased to 20 and 30 wt%. With the exception of one discernible peak shifted to 21.2° for alpha phase PF, TSF/PF spectra lines had the same profile as TSF NCs'. It showed that TSF NCs' in powder and their distribution in the hybrid matrix had the same construct and that the casting process had no impact on crystallographic of TSF NCs'. The intensity and number of peaks change noticeably depending on the ratio of addition to the polymer. TSF/PF nanocomposites have a smaller degree of crystallinity than TSF NCs', suggesting that PF is uniformly distributed in the TSF NCs' formulations as recorded in Table.1.

**Table 1:** The estimated crystallization parameters, crystallize size R, and dislocation density of TSF NCs, PF, 10 TSF/PF, 20 TSF/PF, and 30 TSF/PF nanocomposites.

Sample	SnO_2_	SrSnO_3_	Fe_3_O_4_	PF	SnO_2_	SrSnO_3_	Fe_3_O_4_	PF
	R (nm)	R (nm)	R (nm)	R	δ × 10^−4^	δ × 10^−4^	δ × 10^−3^	δ × 10^−3^
TSF	75	47.68	11.3	…	1.7	4.4	7.83	…
PF	…	…	…	7.2	…	…	…	1.93
10TSF/PF	11.65	…	12.67	3.37	0.73	…	6.25	0.88
20TSF/PF	84.5	29.75	9.53	6.60	1.4	0.113	0.11	0.23
30TSF/PF	76	37.84	6.73	6.41	1.73	6.98	0.221	0.243

To gain a better understanding of the crystallization process, the dislocation density has been determined by applying Formula 2 to indicate the synergistic action of TSF NCs on PF^27^.

2 $$\delta = \frac{1}{{R^{2 } }}$$

The calculated values δ of PF are reduced with the addition of TSF NCs, and this is noticeable at the proportions of 20 TSF/PF. The lattice imperfections appear in the crystal structure as the dislocation in the lattice increases, resulting in an excess of grain boundaries. A small dislocation, minor defects, and a large crystal size may help with the electrical performance of materials.

**Figure 4:**
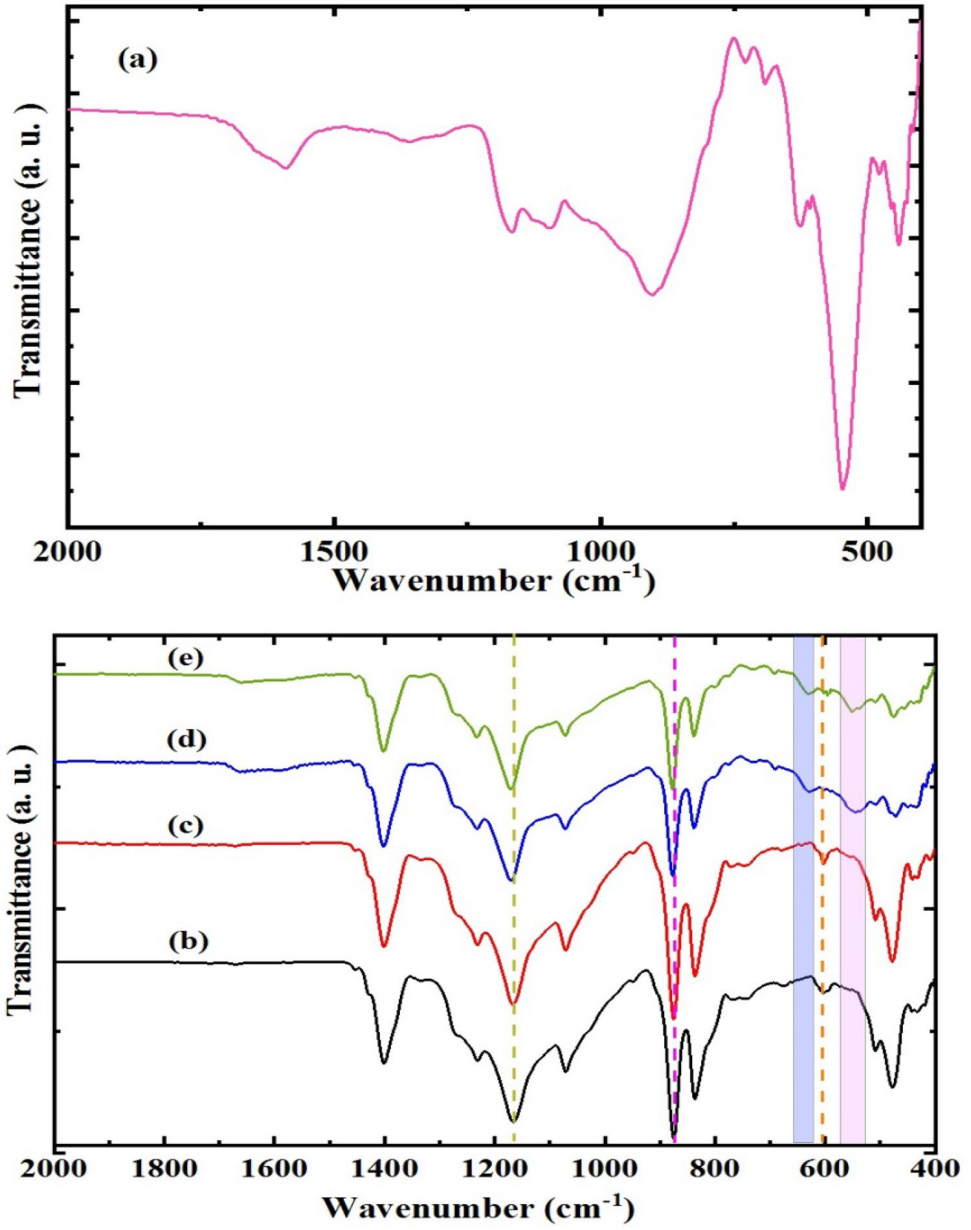
ATR-FTIR of (**a**) TSF NCs, (**b**) PF and PF incorporated with (**c**) 10 wt%, (**d**) 20 wt% and (**e**) 30 wt% of TSF NCs'.

Figure 4 represents FTIR of TSF NCs, PF and PF incorporated with different amount of TSF NCs'. In Fig. 4a, the sharp band at 441 cm^-1^hasattributed to the Sr–O bonding^30^ while the prominent bands ranged from 590 to 490 cm^−1^ are corresponded to the bond vibration of Fe–O and Sn–O^31^. The band located at 625 cm^−1^ is pointed to Fe–O bond in the Fe_3_O_4_ crystalline lattice^32^. Distortion styles of Sn–O bonds in SnO_6_ octahedral otherwise bend of Sn–O–Sn bridges were typically seen at 903 cm^−1^^33^ which confirmed the formation of TSF NCs'.

Figure 4b represents FTIR of PF as shown it has characteristic bands at 1401 cm^−1^ and 1070 cm^−1^ which is related to wagging mode of CH_2_ bond. In the range of 1120–1350 cm^−1^, the CF_2_ group has a strong absorption. The CF_2_ asymmetric stretching vibration is shown at 1231 cm^−1^ and 1167 cm^−1^. The asymmetric vibration of C–C–C bond is seen at 875 cm^−1^ while The CH_2_ rocking vibration is observed at 837 cm^−1^. The CF_2_ bending vibration is seen from 670 to 477 cm^−1^^34–36^.

It can be seen that there is a noticeable variation in the intensities of all bands for PF embedded with various weight percentages of TSF NCs' (Fig. 4b). With an increase in TSF NCs' concentration, there is a shift toward higher frequency in the bands at 1167 cm^−1^ and 875 cm^−1^. Additionally, the detected band at 604 cm^−1^ vanished at higher TSF NC content, and new bands emerged at 630 cm^−1^ and 545 cm^−1^. The interaction and complexation between PF and TSF NCs' were confirmed by the aforementioned results and by XRD data.

**Figure 5:**
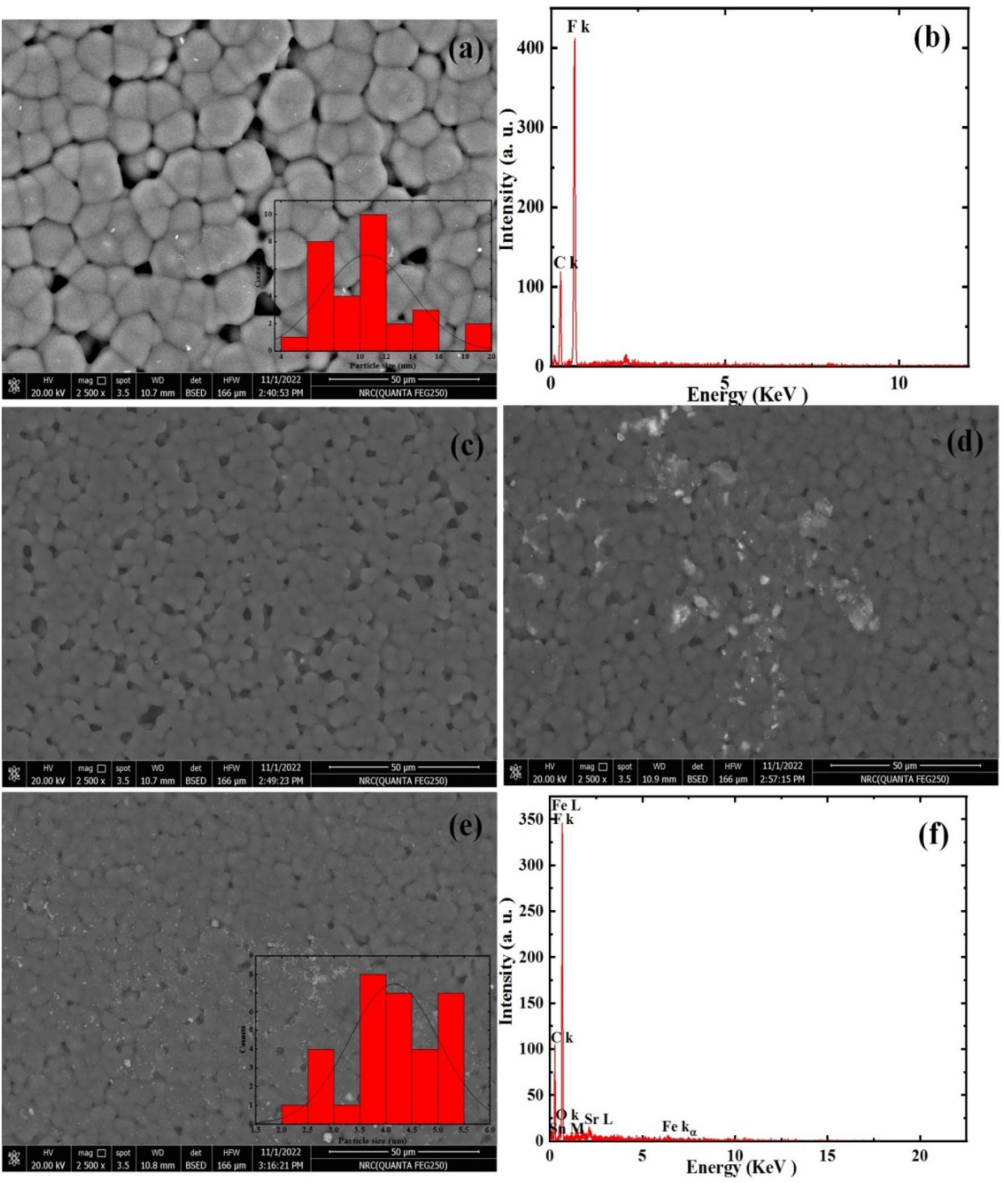
FESEM and EDX of (**a**, **b**) PF and PF incorporated with (**c**) 10 wt%, (**d**) 20 wt% and (**e**, **f**) 30 wt% of TSF NCs'.

Figure 5 represents FESEM of PF and PF incorporated with different amount of TSF NCs'. Figure 5 a revealed the spherulitic microstructure typical of PF, with dimensions between 6 and 19 μm. EDX analysis confirmed the existence of carbon and fluorine components which related to PF polymer (Fig. 5b). When PF/TSF NCs'. were examined, it was discovered that the size of the microstructure had reduced. It was discovered that the spherulitic sizes became ranged between 2 and 5.5 μm (Fig. 5e). This decrease in size can be ascribed to the tremendous electrostatic interactions that exist among the polymer chains besides TSF NCs'. These interactions result in the formation of a well-defined local polar–polar region. EDX analysis approved the existence of C and F, O, Sr, Fe and Sn components which related to the incorporation of TSF NCs' to PF polymer (Fig. 5f). Besides the spherulitic shape that corresponded to PF polymer, there is a small particles distributed randomly on PF with addition of TSF NCs'.

**Figure 6:**
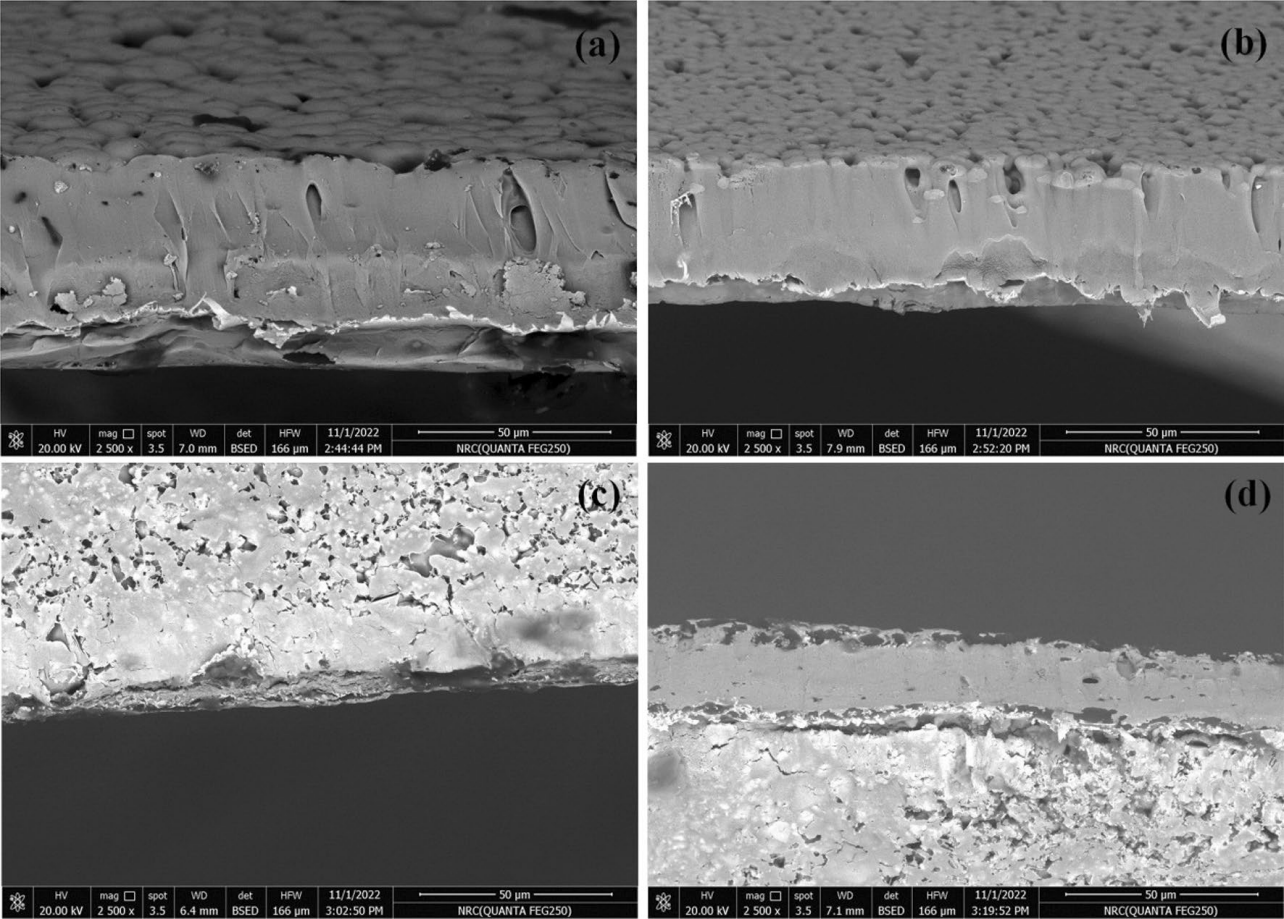
Cross section of (**a**) PF and PF incorporated with (**b**) 10 wt%, (**c**) 20 wt% and (**d**) 30 wt% of TSF NCs'.

Figure 6 illustrates the cross-section area of PF and PF incorporated with different amounts of TSF NCs'. The cross section image of PF shows that there is a porous and loosely linked particle of PF with inner channels with size 6 μm. By increasing the amount of TSF NCs', it was not able to determine the limits between grains and the size of the spherulites for PF/TSF was not measured. This indicated the incorporation of TSF NCs' inside PF porous and changed its surface characteristic.

**Figure 7:**
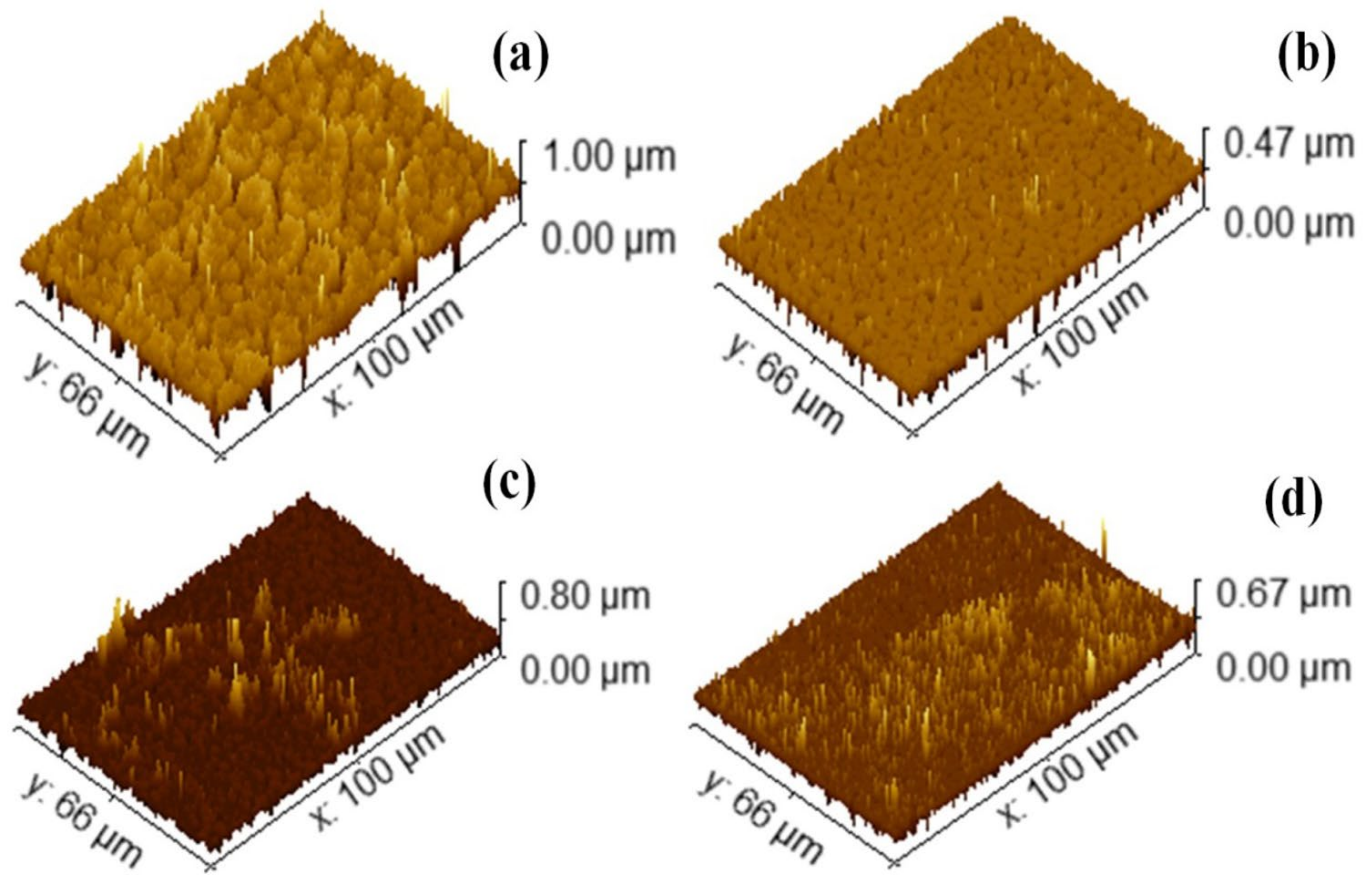
3D images of (**a**) PF and PF incorporated with (**b**) 10 wt%, (**c**) 20 wt% and (**d**) 30 wt% of TSF NCs'.

Figure 7 represents 3D graphs of PF and PF/TSF NCs' using Gwyddion software. As observed the roughness of the surface is decreased by addition of TSF NCs'. The estimated values of root mean square roughness and average roughness of prepared samples were shown in Table 2. It is noticed that both $R_{rms}$ besides $R_{a}$ reduction with adding of TSF NCs' relative to pure polymer. Functional metrics such as skewness (R_s_) and kurtosis (R_k_) were utilized in order to further define the surface roughness. Using R_s_, one can determine the degree to which the profile has symmetrical with respect to the average line. This characteristic is particularly sensitive to the occurrence of infrequent deep valleys and high peaks. A negative value for R_s_ represents a profile with no peaks or significant scratches. A profile that has high peaks or filled-in valleys is represented by a R_s_ value that is positivein order to learn more about the distribution of the spikes above and below the mean line, the R_k_ statistic is used. The surface is planar, and the distribution curve is referred to as platykurtic when R_k_ is smaller than 3. This indicates that the curve has few high peaks and many low valleys. When R_k_ exceeds 3, the distribution curve is said to be leptokurtic since there are proportionally more peaks than valleys^37^. It is observed from Table 2 that PF and 10TSF/PF samples has negative Rs values implying that the valleys were more prominent than the peaks. For 20TSF/PF and 30TSF/PF samples, R_s_ has a positive value which means that they have a spiked morphology. For R_k_ values, only 20TSF/PF sample less than 3 which means that it has low valleys and low peaks compared to other samples. It is critical to understand that the majority of engineering surfaces have interfacial ($R_{rms}$/$R_{a}$) that are approximately 1.31^37^. Table 2 shows percentages of it’s $(R_{rms}$/$R_{a}$) that are comparable to the majority of engineering surfaces (1.31). The extensive research discussed above suggests that it was effective to generate TSF NCs' nanocomposites with shrinkage surface roughness.

**Table 2:** Values of $R_{rms}$, $R_{a}$, R_s_ and R_k_ of PF and PF/TSF NCs nanocomposites.

Sample	$R_{rms}$ (nm)	$R_{a}$ (nm)	R_s_	R_k_	$R_{rms} /R_{a}$
PF	119.3	84.9	− 1.52	4.1	1.405
10TSF/PF	43.3	31.1	− 0.63	1.6	1.392
20TSF/PF	50.1	27.1	1.61	10.2	1.848
30TSF/PF	46.2	39.2	0.83	3.9	1.178

### UV–Vis characterization

**Figure 8:**
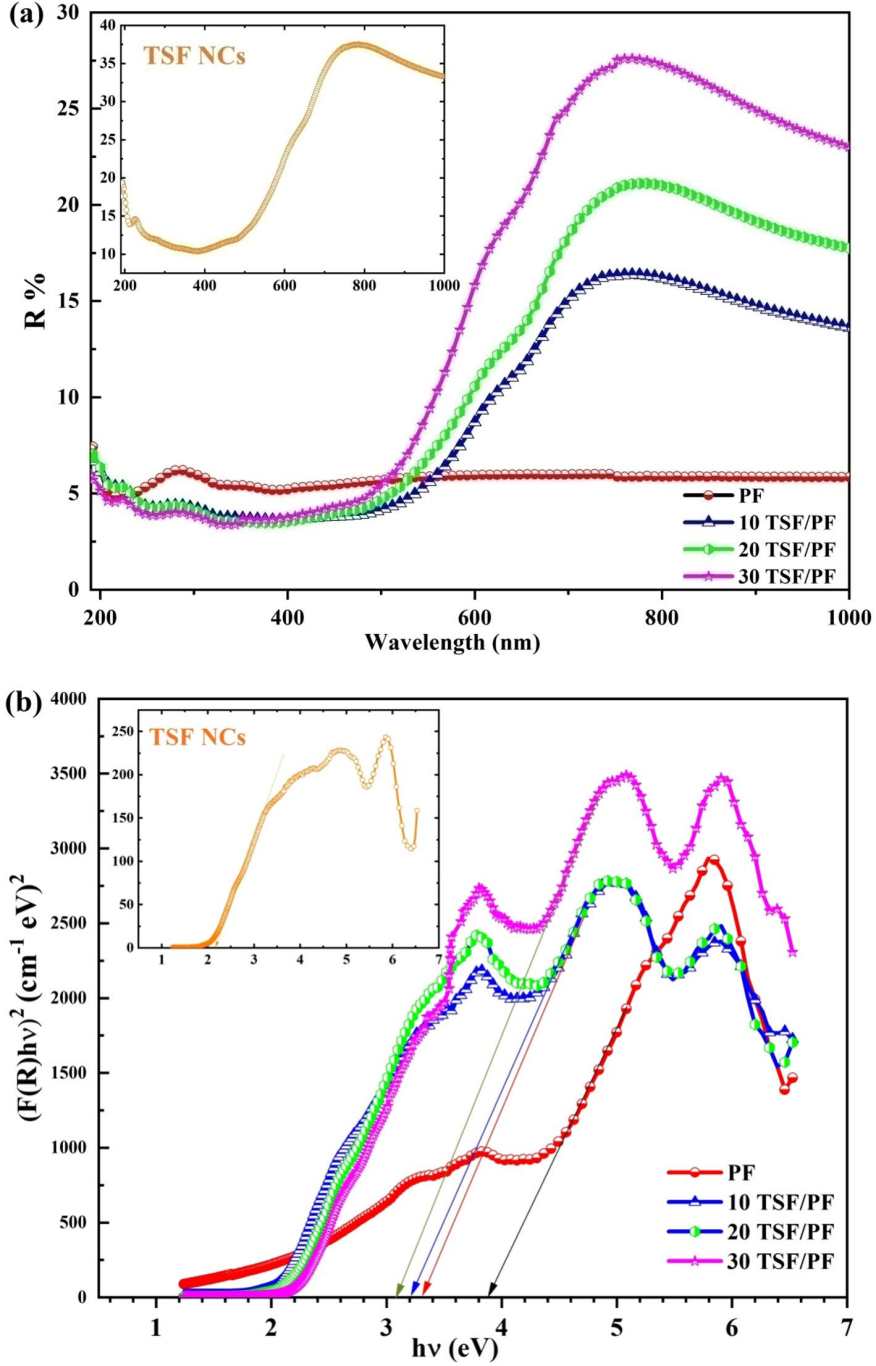
(**a**) Diffuse reflectance and (**b**) (F(R)hν)^2^versus hν of TSF NCs', PF and, PF incorporated with (**b**) 10 wt%, (**c**) 20 wt% ,and (**d**) 30 wt% of TSF NCs'.

Figure 8a represents DRS of TSF NCs' and PF incorporated with different amount of TSF NCs as a function of wavelength. As observed the reflection values tend to be increased at 400 nm ≤ λ ≤ 800 nm. It is interesting to note that the effect of inserting TSF NCs' contents in PF polymer leads to an increase in reflectance values. A high reflectance indicates that light is unable to penetrate thin film samples with any significant amount of effectiveness. This is an indicator that polymeric thick matrix that have been doped with a high ratio of TSF NCs' have a high competence in avoiding the absorption of radiation in the visible spectrum range. An important step forward in the production of a new generation of optical instruments was taken when it was discovered that the reflectance of TSF NCs' could be adjusted and tuned by impressive proper procedure conditions and doping levels on the NPs.The optical band gap energy (E_g_) is calculated using Kubelka–Munk function^38^:

3 $$F\left( R \right) = \frac{{\left( {1 - R} \right)^{2} }}{2R}$$

where R is the reflectance and F(R) is the reflectance coefficient. Figure 8b shows the relation between (F(R)hʋ)^2^against photon energy (hʋ). As observed, values of band gap energy are estimated by extrapolating the straight line of the curve to zero in the hʋ axis. The values of E_g_ are 2.1 eV, 3.90 eV, 3.31 eV, 3.20 eV and 3.07 eV for TSF NCs', PF, 10 wt% TSF NCs', 20 wt% TSF NCs', and 30 wt% TSF NCs', respectively. In comparison to the optical energy bandgap of pure PF, it was observed that the optical energy bandgap of nanocomposites decreased as the weight of TSF NCs' increased in the nanocomposites. This pattern was noticed because nano-filler particles have the ability to organize themselves in the polymer template, which leads to the construction of conductive routes through which electron hopping can take place. This ability was responsible for the observation of the trend. A circumstance like this one causes a shift in both the VB and the CB, as well as an improvement in carrier-carrier interaction. The bandgap is narrowed because there is a significant concentration of carriers in both the VB and the CB. The shift in the bandgap demonstrates that the inclusion of the nano-filler modifies the energy levels of the pure PF. This creates the localized states in the forbidden band, which may cause the Fermi position to alter. Additionally, these states serve as recombination and trapping sites. Hence, as a result of low energy transitions, the nanocomposites' optical conductivity was improved^39^.

**Figure 9:**
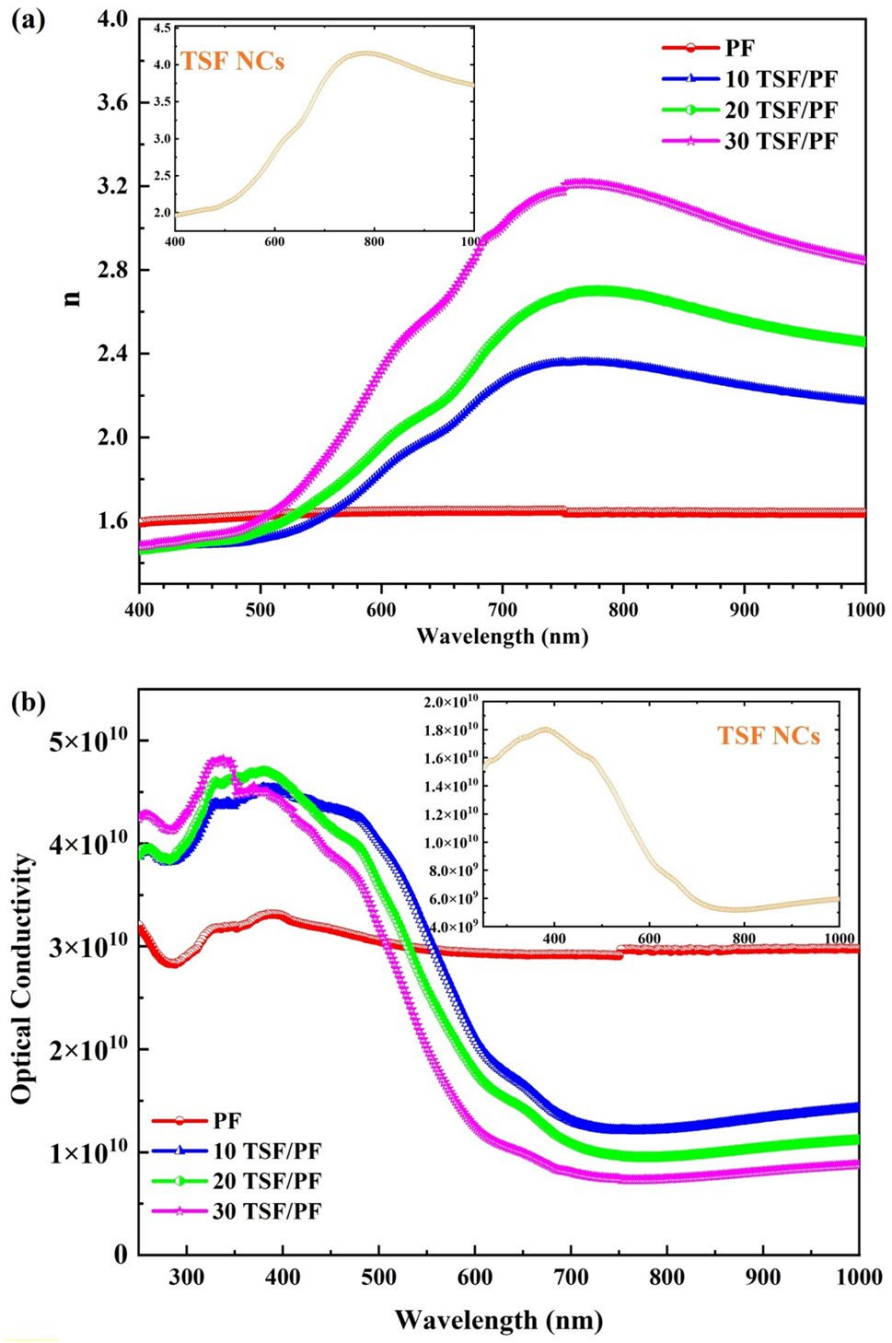
(**a**) Refractive index (n) and (**b**) optical conductivity against wavelength (λ) of TSF NCs', PF and TSF/PF nanocomposites.

When building optical constituents for optical instruments, it is absolutely necessary to conduct precise measurements of the refractive index (n) of the films being used. The following is one way to explain what it looks like in terms of reflectance (R) as illustrated in Fig. 9a^40^:

4 $${\text{n}} = \frac{{1 + {\text{R}}^{{{\raise0.7ex\hbox{$1$} \!\mathord{\left/ {\vphantom {1 2}}\right.\kern-0pt} \!\lower0.7ex\hbox{$2$}}}} }}{{1 - {\text{R}}^{{{\raise0.7ex\hbox{$1$} \!\mathord{\left/ {\vphantom {1 2}}\right.\kern-0pt} \!\lower0.7ex\hbox{$2$}}}} }}$$

It has been found that the value of n increases with the addition of TSF NCs', indicating that the films get denser as the refractive index rises.As a direct consequence of this, the speed at which light travels through thin films made of PF/TSF nanocomposite is greatly slowed down. The aggregation of smaller nanoparticles into bigger clusters may be responsible for the higher refractive index values of the TSF/PF nanocomposite. The elevated n values of the investigated nanocomposite are also encouraging and imply that these films might be used for potent optical confinement. The optical conductivity (σ_opt_) has determined using the following formula^41^:

5 $$\sigma_{{opt = \frac{nF\left( R \right)C}{{4\pi }}}}$$

where C is the speed of light and αis absorbance coefficient and it is proportional to *F*(*R*)^42^. Figure 9b represents the relation between σ_opt_ against wavelength of incident light. The optical conductivity of the nanocomposite films that were fabricated has higher values in the UV area because the TSF NCs in this region absorbs more of the incident photon energy than it does in the visible region. This is the reason why the optical conductivity values in the UV region are higher. Figure 9b shows that the optical conductivity in the visible area drops gradually at first and then stays at the same level. A meager value for optical conductivity in the visible region indicates that there is very little electronic excitation of photons that are incident in that region. The PF/TSF NCs films that were developed have the potential to be used as UV absorbers due to their high capacity for absorbing UV rays. The graph shows that increasing the amount of TSF in nanocomposite results in agrowth in the optical conductivity of the material compared to pristine PF and TSF NCs'.

### Dielectric properties

**Figure 10:**
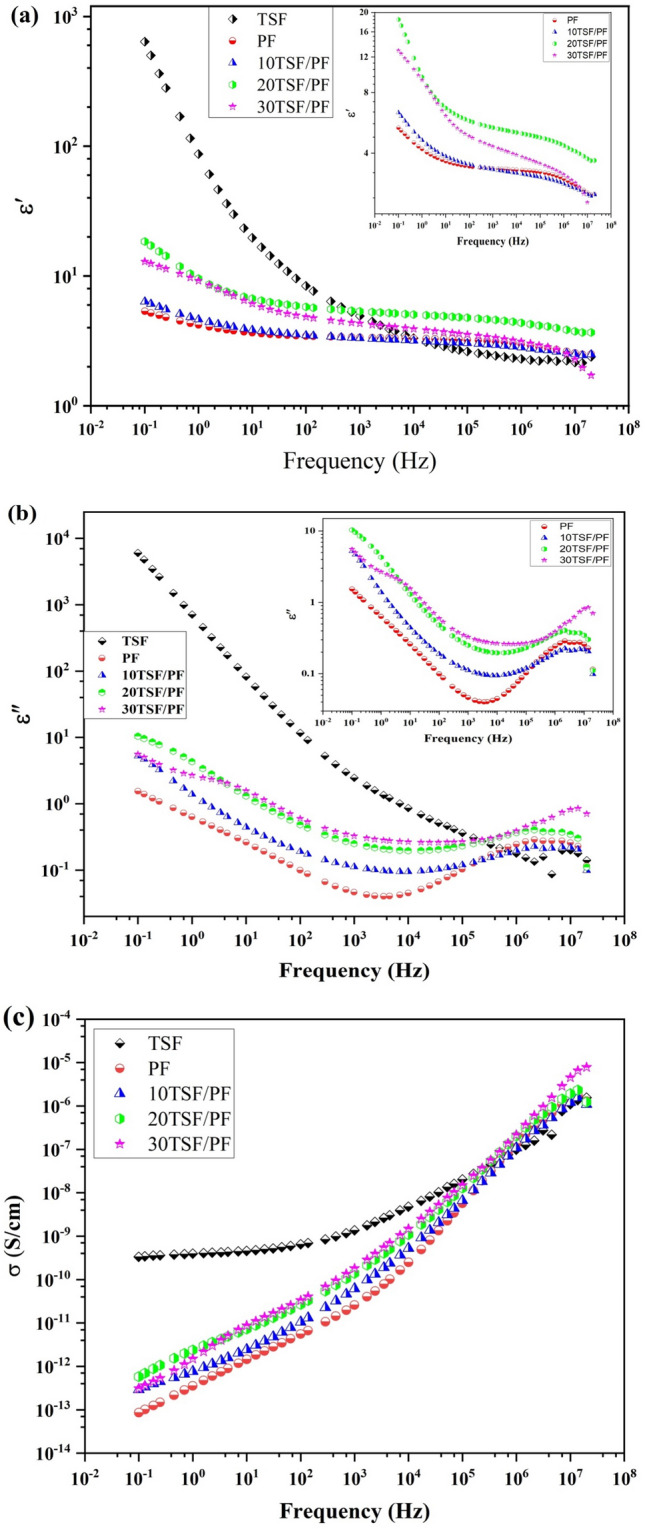
The frequency-dependent plot of the (**a**, **b**) ε′ and ε″ sections of dielectric permittivity and (**c**) conductivity plot of TSF NCs', PF, and TSF/PF nanocomposites.

Interface characteristics between the polymer matrix and nanoparticles have a major impact on the dielectric performance of complexes. Improving the dielectric constant of the composites is a result of the polymer matrix and conductive nanoparticles, which both encourage the relocation and accumulation of the charge carriers at the filler-matrix boundary’s due to the differing relaxation durations of the phases. Figure 10 depicts the variation of dielectric constant and loss vs. frequency for PF and PF incorporated with various proportions of TSF NCs'. The permittivity is significantly reduced in the higher frequency range, while the dielectric constant rises with decreasing frequency (10^4^–10^6^ Hz). This is due to the composites' dipole's inability to keep up with the external field's frequency variations. All of the samples' relative permittivity’s exhibit a similar frequency relationship. Due to the electronegative fluorine atoms in its molecular structure and the interface polarization that is seen at the PF's alpha and beta phase interface, it is widely known that PF has a significant dipole moment. Because the average electric field in the polymer matrix increases with the addition of TSF NCs' to PF, the relative permittivity rises. All composite films have relative permittivity’s that are higher at 1 Hz than the clean PF film (4.3). In addition to inducing interfacial polarization, TSF NCs added to the PF matrices further raise relative permittivity. As the density of TSF NCs dipoles increases, so does the dipolar polarity.The relative permittivity of the PF composite is further increased by the introduction of TSF NCs' as a result of MWS (Maxwell–Wagner–Sillars) polarization.

The change of dielectric loss versus frequency is displayed in Fig. 10b. Because the conductivity loss is reduced when TSF NCs' are introduced into PF, it can be seen that the dielectric loss dropped from 0.1 to 10^3^ Hz because interfacial polarization and dipole orientation polarization were parts of the gradual depolarization. As a result, they successfully prevent ion conduction within the composites, and the interface polarization does not fluctuate on its own in response to frequency changes.Due to the relaxing process and gradually growing conductivity loss caused by TSF/PF nanocomposites, the dielectric loss increased from 10^3^ to 10^7^ Hz.This might be seen as a consequence of the C–F dipole polarization process that took place in the PF matrix. The torque produced by the C–F dipole, when subjected to an applied electric field, causes the macroscopic dipole moment to deviate from zero. In turn, this results in Debye relaxation during the polarization process of the dipole orientation, which raises the dielectric loss at large frequencies. In general, the conduction loss and the dielectric polarization loss of dielectric materials are primarily responsible for the dielectric loss. Other contributing factors include the migration of space charges and the motion of molecular dipoles^43,44^.

Because an increase in carrier concentration and mobility also results in an increase in conductivity, TSF/PF nanocomposites have a higher dielectric constant and dielectric loss. Molecular polarizability of polymer nanocomposites was found to be the primary determinant of the polymer's dielectric constant and dielectric loss in a previous investigation. By altering the kind and number of polarizable groups present in the polymer matrix, this molecule polarizability can be changed.

The association between the conductivity and frequency at room temperature is depicted in Fig. 10c for PF and PF incorporated with different amounts of TSF NCs'. As shown in Fig. 10c, as the frequency lowers, an increasing quantity of charge builds up at the electrode-sample interface, which causes the conductivity of measured samples to increase. This leads to a reduction in the number of mobile ions, which in turn causes the conductivity to decrease at low frequencies. The increased mobility of charge carriers that occurs at larger frequencies contributes to agrowth in conductivity in the material^45^. After doping with TSF NCs', there was an increase in the electrical conductivity, because the incorporation of TSF NCs' into PF polymer results in an increase in the number of electrons and, as a consequence, the blend's conductivity. This is accomplished by expanding the number of pathways along which electrons can travel.

**Figure 11:**
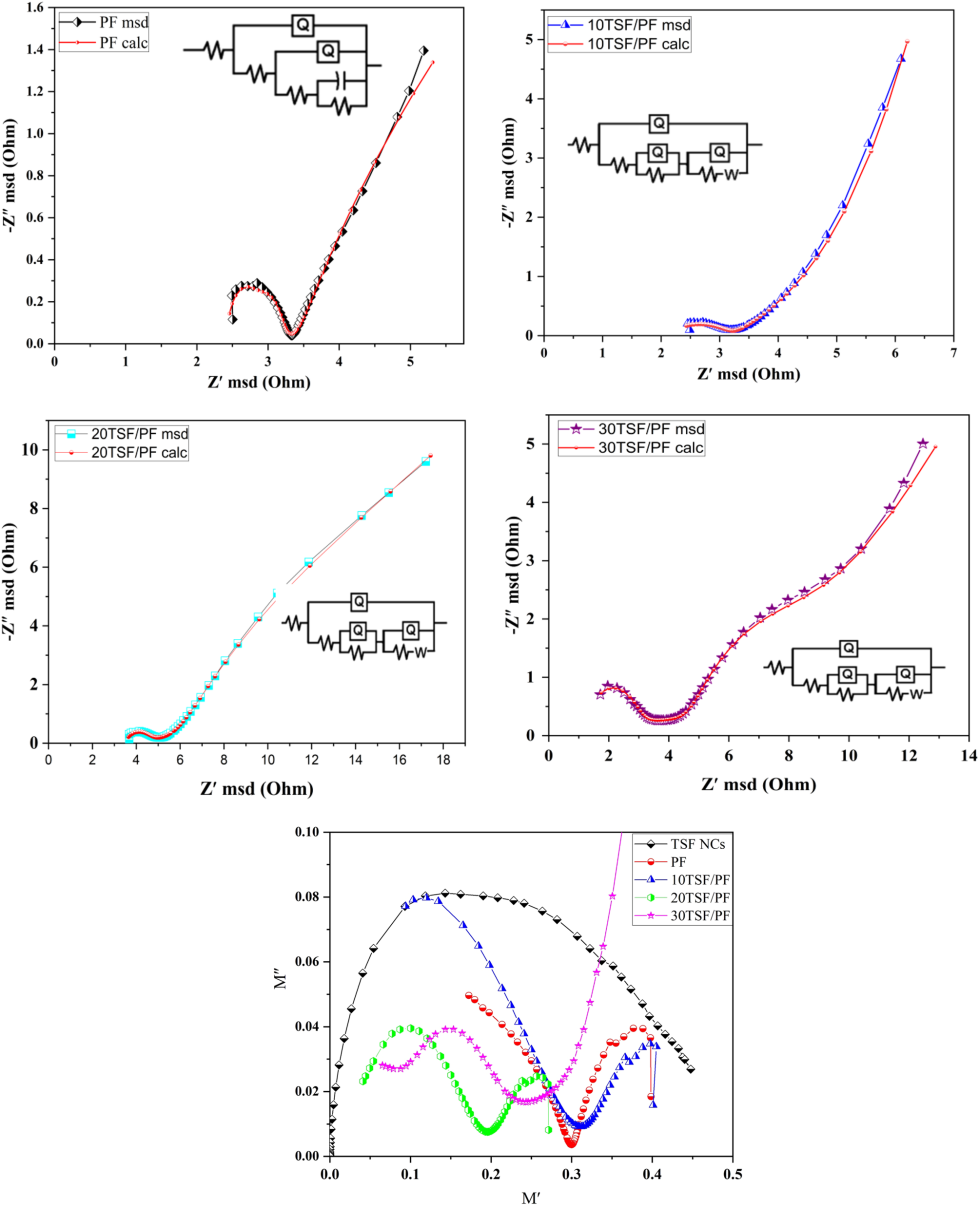
(**a**) The impedance spectra, and (**b**) the complex modulus plots of TSF NCs', PF, and TSF/PF nanocomposites.

The great density of imperfections, like as deficiencies and dangling bonds, that are located at these grain boundaries could have a big effect on how these materials move.As shown in Fig. 11a^46^, impedance spectroscopy(Nyquist-plot) makes it straightforward to comprehend the functions that grains and grain boundaries perform that measured by Z sim demo software. The material under investigation's impedance data revealed a depressed semicircle and position change, the radius of which shrank as the amount of TSF NCs' incorporated into PF increased. The decrease in resistance (or increase in conductivity) with TSF/PF indicates semiconductor behavior if the semicircles' widths shrink as the TSF NCs' amount rises. Without centers on the actual axis, the dip was seen for all semicircles at all composites except 30 TSF/PF. Due to the distribution of relaxation times within the bulk material, such non-Debye-type behavior was taken into consideration. Such materials' optimum impedance spectra display a semicircle. The impedance contribution from the grain, grain boundary, and electrode-material contact can be taken into account when an oxygen-deficient perovskite and oxide semiconductor are evaluated as an electrolyte. As a result, three semicircular arcs that correspond to the grain may be anticipated.

The semicircle at high frequency denotes the effect of the grain (bulk), as well as the semicircle at lower frequencies denotes the grain boundary. The impedance spectra could be accurately recreated within the experimental uncertainty, and this is a crucial point to remember. This means that other possible explanations for the lower frequency loop are ruled out, including reactions between the electrodes and the surfaces of the materials^47,48^.

The existence of multiple relaxation processes occurring at the same time or a distortion in the grain boundary could be the origin of the grain boundary's less-than-ideal behavior as depicted in Fig. 11b.

The Cole–Cole plot (M″ vs. M′) separates relaxation impacts from grains (conducting zones) and grain boundaries (resistive slabs) in components better than the Nyquist plot of impedance (Z″ vs. Z′)^49^. The complex modulus graphs for TSF NCs', PF, 10 TSF/PF, 20 TSF/PF, and 30 TSF/PF nanocomposites are displayed in Fig. 11b. It is obvious that one, two, and three well distinct semicircles have been seen relying on the grain, interface and grain boundary and also electrode polarization. The widening of the peak shows the dispersion of the relaxation with a constant relaxation time distribution. Peaks in M″ and M' also serve as a clear indicator of the dielectric relaxation process itself^49–52^.It has also been observed that when nanocomposites proportions increase, the grain semicircle's intercept on the M' axis begins to decrease, implying an increase in capacitance.

### Magnetic explorations

**Figure 12:**
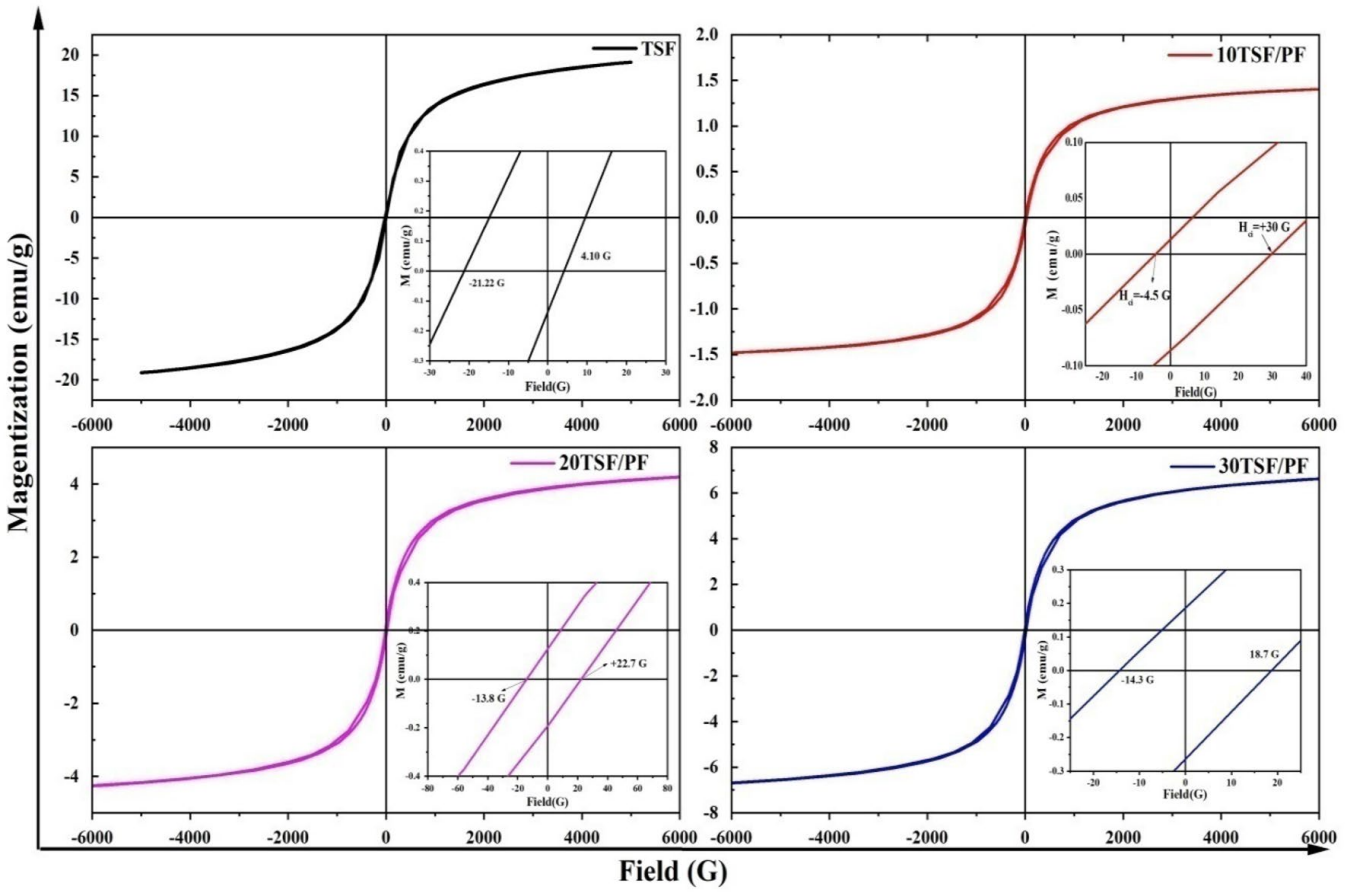
The M–H loop, of TSF NCs', and TSF/PF nanocomposites.

Due to their vast range of uses in information storage, transportation, and biomedical devices, magnetic materials are extensively dispersed^52^. Figure 12 depicts the M-H loops for TSF NCs' and TSF/PF nanocomposites at 300 K. The majority of the magnetic characteristics are entirely reliant on the size, morphology, and magnetic domain state^53,54^. Fe_3_O_4_ nanoparticles are incorporated in SnO_2_/SrSnO_3_ composites and exhibit ferromagnetic behaviour with a remarkably small coercivity and retentivity of 12.66 G and 0.38 emu/g, as can be seen in Fig. 12 as an inset, and saturation magnetization of 19 emu/g that was confirmed from the XRD and EDX results. The separation of super-exchange bonds closer to the surface evidences a canted spin formation even as the magnitude of the Fe ionic species continues to decrease^55,56^.Moreover, because PVDF is non-magnetic, it is associated with a lower magnetic sensitivity of nanocomposites when contrasted to Fe_3_O_4_, as shown in Fig. 12.

However, because of the increase in TSF NCs' content, ferromagnetic behaviour, i.e., Ms. and Mr., improves at 30 TSF/PF, particularly in comparison to 10 TSF/PF. Coercivity, besides, controls a ferromagnetic substance's resistance to demagnetization. A relatively similar coercivity for all specimens in this work suggests that the coercivity is solely reliant on magnetite nanoparticles and that the nanoparticles are uniformly dispersed in the polymer matrix^57,58^.

**Figure 13:**
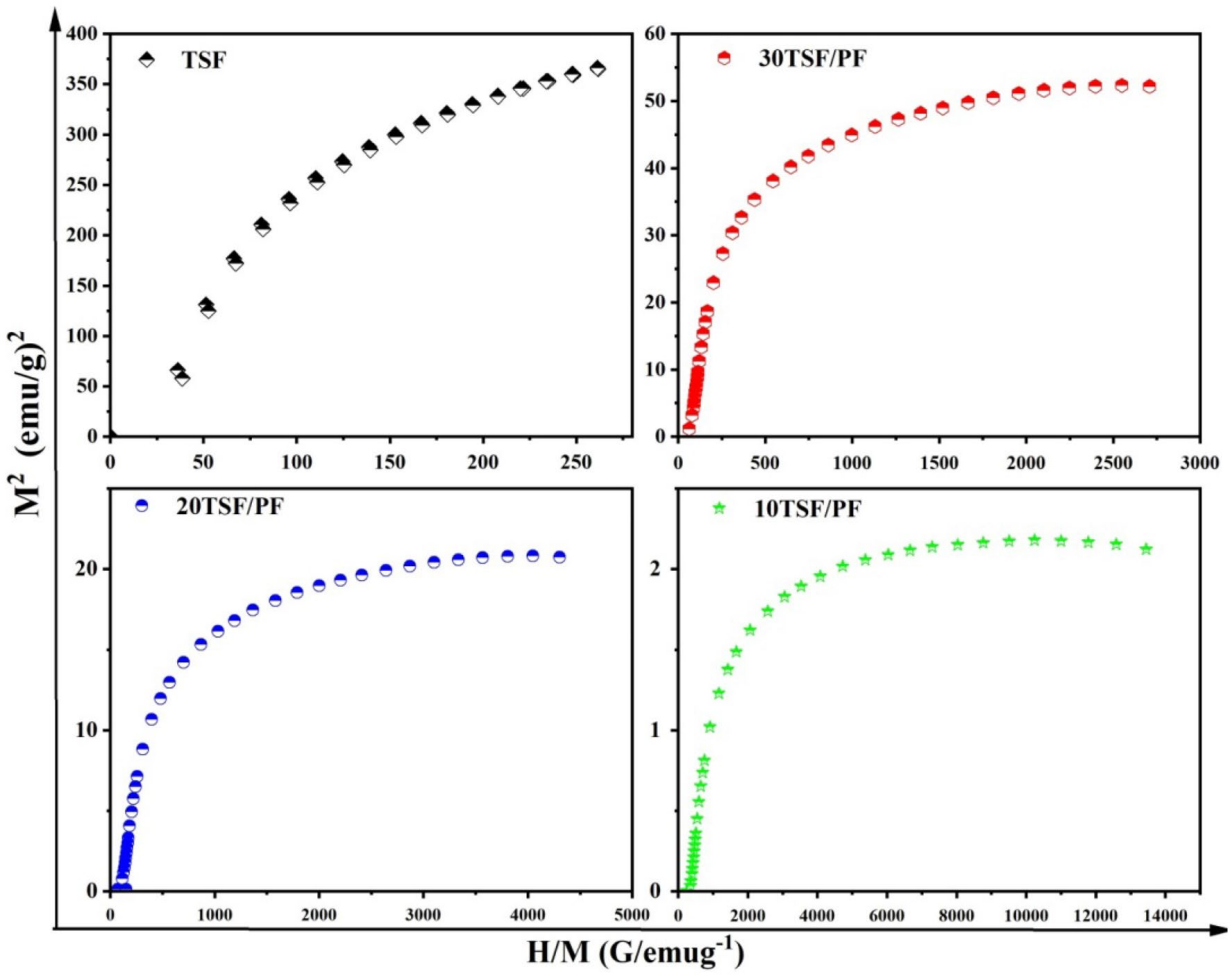
Arrott-Belov-Kouvel (ABK) graphs of TSF NCs', and TSF/PF nanocomposites.

For a detailed explanation of magnetic behaviour, we plotted perfectly recognized Arrott–Belov–Kouvel (ABK) graphs for the samples, as illustrated in Fig. 13.The ABK figures (M^2^ vs. H/M plots) at room temperature for the entire sample display slightly curved curvature with finite spontaneous magnetization, suggesting the ferromagnetic phase of the substances, which significantly reduced with the amalgamation of nonmagnetic PF phase, as can be seen in Table 3.

**Table 3:** Magnetic parameters of TSF NCs,TSF/PF nanocomposites , saturation magnetization (MS), remnant magnetization (Mr), coercively (Hci), and spontaneous magnetization(SPM).

Sample	M_s_ (emu/g)	M_r_ (emu/g)	H_ci_ gauss (G)	SP_M_ (emu/g)^2^
TSF	19	0.37	12.66	150
10 TSF/PF	1.515	0.049	17.05	1.9
20 TSF/PF	4.59	0.125	18.21	16
30 TSF/PF	7.27	0.186	16.51	34

The magnetic saturation value of the composites increased as the TSF NCs' concentration increased, implying that magnetic nanoparticles influenced the ferromagnetic behaviour observed in the composites for promising photocatalyatic magnetic separations.

## Conclusions

TSF/PF nanocomposites were effectively prepared using facile assembly, co-precipitation, and drop casting techniques. XRD and ATR-FTIR affirmed the creation of TSF NCs'. Also, the elemental composition of Sn, Sr, Fe, and oxygen in EDX analysis approved the formation of TSF NCs'. HRTEM images shows the appearance of elongated nanorods, nanocubes and semispherical shape with SAED image confirmed the formation of polycrystalline substance. The miscibility and distribution of TSF NCs' inside PF polymer are supported by XRD and ATR-FTIR. The shifting in the bands at 1167 cm^−1^ and 875 cm^−1^and appearance new bands at 630 cm^−1^ and at 545 cm^−1^ confirmed the interaction between the polar group of TSF NCs' and PF. The integration of TSF NCs' into porous PVDF material resulted in a change in the surface characteristic of the material, and reduction in the surface's roughness. As the percentage of TSF NCs' in the nanocomposites increased, the optical energy band gap reduced. Due to the capacity of nano-filler particles to arrange themselves in the polymer matrix, conductive paths can be built, allowing electron hopping to occur. The valence and conduction bands shifted and the carrier-carrier interaction is enhanced which leading to increasing the optical conductivity.After doping with TSF NCs', the electrical conductivity is increased. This is accomplished by expanding the number of pathways along which electrons can travel.The ferromagnetic behavior that was observed in the composites was likely impacted by magnetic nanoparticles since the magnetic saturation value of the composites increased as the amount of TSF NCs' increased. These TSF/PF nanocomposites support optical, electric, and magnetic properties in promising magneto-optoelectronic implementations.

## Data Availability

The datasets produced and/or analyzed during the current study are available upon reasonable request from the corresponding author.
